# Association Between Body Mass Index and Cancer Screening Adherence Among Latinas in the United States and Puerto Rico

**DOI:** 10.1089/whr.2021.0153

**Published:** 2022-05-31

**Authors:** Maira A. Castaneda-Avila, Jonggyu Baek, Mara M. Epstein, Sarah N. Forrester, Ana P. Ortiz, Kate L. Lapane

**Affiliations:** ^1^ Division of Epidemiology, Department of Population and Quantitative Health Sciences, University of Massachusetts Chan Medical School, Worcester, Massachusetts, USA.; ^2^ Division of Biostatistics and Health Services Research, Department of Population and Quantitative Health Sciences, University of Massachusetts Chan Medical School, Worcester, Massachusetts, USA.; ^3^ Meyers Health Care Institute, a Joint Endeavor of the University of Massachusetts Chan Medical School, Fallon Health, and Reliant Medical Group, Worcester, Massachusetts, USA.; ^4^ Division of Geriatric Medicine, Department of Medicine, University of Massachusetts Chan Medical School, Worcester, Massachusetts, USA.; ^5^ University of Puerto Rico, Comprehensive Cancer Center, San Juan, Puerto Rico.; ^6^ Graduate School of Public Health, Medical Sciences Campus, University of Puerto Rico, San Juan, Puerto Rico.

**Keywords:** cancer, health disparities, obesity, colorectal cancer, epidemiology

## Abstract

**Background:**

Research on the role of body size on cancer screening is mixed with few studies among Latinas in the United States. We evaluated the association between body size and cancer screening adherence among Latinas living in Puerto Rico and the rest of the United States.

**Methods:**

We conducted a cross-sectional study using 2012–2018 Behavioral Risk Factor Surveillance System data among Latinas 50–64 years of age (*n* = 16,410). Breast, cervical, and colorectal cancer screening (guideline adherent: yes/no), height and weight were self-reported. Prevalence ratios (PRs) derived from Poisson models were estimated for each cancer screening utilization for Puerto Rico versus rest of the United States by body mass index (BMI) category.

**Results:**

Nearly a quarter of women lacked adherence with breast and cervical cancer screening and 43.6% were nonadherent to colorectal cancer screening. Latinas with BMI ≥40.0 kg/m^2^ in both groups were more likely to lack adherence to cervical cancer screening than women with BMI 18.5–24.9 kg/m^2^. For those with BMI ≥40.0 kg/m^2^, Latinas in Puerto Rico were more likely to lack adherence to colorectal cancer screening recommendations than Latinas living in the rest of the United States (adjusted PR: 1.38; 95% confidence interval = 1.12–1.70).

**Conclusions:**

The role of body size in cancer screening utilization among Latinas differs in women living in Puerto Rico versus in the rest of the United States and varies by cancer type. Understanding Latinas' experience can inform culturally adapted interventions to promote cancer screening.

## Introduction

Breast, cervical, and colorectal cancer are leading sites of new cancer cases and deaths among Latinas (Hispanic/Latino women) in the United States.^1^ For these cancer types, routine cancer screening tests are recommended.^2–4^ Despite policy interventions such as The Affordable Care Act and many state laws, screening rates remain suboptimal.^5^ Hispanic/Latinos encompasses a heterogeneous group; this population includes people from Mexico, Central and South America, Puerto Rico, Cuba, and other countries of origin. Even when countries of origin appear to be similar (*i.e.*, same language), each group may have differing dialects, traditions, migration status, subcultures, and life experiences.^6^

Puerto Rico is the largest United States territory with ∼3.2 million people, of whom 99.7% are Hispanic.^7^ Puerto Ricans constitute the second-largest Hispanic population living in the United States.^8^ The screening rates for cervical (84%), breast (79%), and colorectal (59%) cancer among Latinas in the United States^9^ were similarly suboptimal when compared with screening rates for cervical (83%), breast (73%), and colorectal (58%) cancer in Puerto Rico.^10^ Despite these similarities, few studies have explored factors associated with receipt of cancer screening tests for Latinas living in these different contexts.

One factor to consider in relation to receipt of screening tests is body size. Obesity and overweight are highly prevalent among Latinas in the United States. In 2018, Latinas were 20% more likely to be overweight than non-Hispanic white women.^11^ The evidence for the association between body size and cancer screening rates among women is mixed.^12^ Women who are overweight or obese are less likely to comply with cervical cancer screening recommendations,^13^ but for breast and colorectal cancer, the association is less consistent. Some studies show women with larger body size were less likely to report guideline-concurrent screening for breast and colorectal cancer,^14–16^ whereas other studies report no association.^17,18^ These findings may differ by race/ethnicity, with less consistency noted for non-Hispanic black women.^12^

The negative attitudes and stereotypes that some health care providers have about people living in larger bodies influence patient's perceptions, judgment, interpersonal behavior, and decision making.^19^ These attitudes can affect the care they provide, causing stress and care avoidance, mistrust of physicians, and poor adherence among obese patients.^20^ In addition, ideas about what constitutes healthy and acceptable body types is highly influenced by culture. For Latinas, data on the association between body size and cancer screening are scant. Although historically Puerto Ricans value larger body types, due to a food scarce environment, negative connotations associated with larger body sizes have begun to increase.^21^

We aimed to evaluate the association between body mass index (BMI) and screening for breast, cervical, and colorectal cancer among Latinas living in Puerto Rico versus Latinas living in the rest of United States.

## Methods

This study used publicly available deidentified data and as such was not considered human subjects research and was exempt from Institutional Review Board review.

### Data source

Data were combined from the 2012, 2014, 2016, and 2018 Behavioral Risk Factor Surveillance System (BRFSS)^22^ to increase the sample size needed to make the subgroup analysis among Latinas living in the United States and Puerto Rico possible. The cancer screening module was fielded in all jurisdictions in these 4 years. Participants were asked separate questions regarding whether they ever had a mammogram, a Papanicolaou (pap) test and a blood stool test, sigmoidoscopy, and/or colonoscopy. The BRFSS also includes questions regarding the last time participants had each cancer screening.

The BRFSS is a telephone survey that has continuously collected data on health-related behaviors, chronic health conditions, and use of preventive services in all 50 states, the District of Columbia, Guam, Puerto Rico, and the U.S. Virgin Islands. The multistage sampling design of households using random digit dialing allows the BRFSS to provide a nationally representative sample of the civilian, noninstitutionalized U.S. population when appropriate weights are applied analytically.

The median survey rate for all states, territories, and Washington, DC, was 45.2% (range: 27.7% −60.4%) in 2012, 47.0% (range: 25.1% −60.1%) in 2014, 47.0% (range: 30.7% −65.0%) in 2016, and 49.9% (range: 38.8% −67.2%) in 2018.^22^ In Puerto Rico, the response rate was 58.2% in 2012, 58.5% in 2014, 55.6% in 2016, and 52.7% in 2018.^22^

The U.S. Preventive Services Task Force (USPSTF) currently recommends: (1) breast cancer screening among women 50 to 74 years old [biennial mammogram]^2^; (2) cervical cancer screening among women 30 to 65 years old (cervical cytology alone [every 3 years]; high-risk human papilloma virus test [hrHPV] alone [every 5 years] or hrHPV testing in combination with cytology [every 5 years])^4^; (3) colorectal cancer screening among adults 45 to 75 years old (annual high-sensitivity guaiac-based fecal occult blood test [FOBT] or fecal immunochemical test [FIT]; stool DNA-FIT [every 3 years]; computed tomography colonography or flexible sigmoidoscopy [every 5 years]; colonoscopy [every 10 years]; or flexible sigmoidoscopy [every 10 years] with annual FIT).^3^

Based on USPSTF recommendations, we selected women between ages of 50 and 64 years for this analysis, as they should have regular screenings for breast, cervical, and colorectal cancer. We excluded those who (1) did not identify as Hispanic (responding “no,” “Do not know, not Sure” or “refused to answer” the question: “Are you Hispanic or Latino?” in BRFSS 2012; Hispanic, Latino/a, or Spanish origin calculated variable in BRFSS 2014, 2015, and 2016); (2) had a personal history of cancer (cancer screening recommendations varied among cancer patients); or (3) were missing data on BMI or cancer screening. Our final study population included 16,410 Latinas 50–64 years of age (Supplementary Fig. S1).

### Cancer screening adherence

Cancer screening adherence was defined following USPSTF recommendations. (1) breast cancer screening: women who had mammography in the past 2 years (adherent) versus women with no history of mammography or those with mammography greater than 2 years (no adherent), (2) cervical cancer screening: women who reported having had a pap smear in the past 3 years (adherent) versus women with no history of pap smear or those reporting a pap smear greater than 3 years ago, and (3) colorectal cancer screening: women who received one or more of the following recommended tests: FOBT annually, sigmoidoscopy in the last 5 years with FOBT in last 3 years, or a colonoscopy in the last 10 years.

Previous studies have found high sensitivity (∼90%) and low specificity (∼50%) for self-reported cancer screening tests.^23,24^

### Body mass index

BMI was calculated using self-reported weight in kilograms (kg) and height in meters (m). We categorized BMI using the Centers for Disease Control and Prevention cut points (<18.5, 18.5–24.9, 25–29.9, 30–34.9, 35–39.9, and >40 kg/m^2^).^25^ Although there may be concerns about under-reporting of weight in phone surveys, weight is generally underestimated by only five pounds.^26^ A strong concordance between self-reported and measured height, weight, and BMI exists, and strong to near-perfect agreement in classification of self-reported weight status and measured central adiposity has been reported.^27^ Thus, BMI is a valid indicator of weight status, although not necessarily health.^28^

### Confounders

Based on the Andersen Model of Health Services Use,^29,30^ different predisposing, enabling, and need factors were evaluated as potential confounders of the association between BMI and screening adherence.

Predisposing factors included age, marital status (married; divorced/living together; divorced/separated; widowed/never married), educational attainment (less than high school; high school diploma; some college or technical school; college graduate), and employment status (employed/homemaker; unemployed/unable to work; retired; student).^31^

Enabling factors included income (≤$35,000, >$35,000, median income of the sample); health insurance coverage (any coverage vs. no coverage), having a personal doctor or health care provider (yes/no), having had a routine checkup within the past 12 months (yes/no), and reported financial barriers to health care access (yes/no).^32,33^

Need factors included self-reported general health (excellent; very good; good; fair/poor); self-reported chronic conditions, which included cardiovascular disease, asthma, diabetes (excluding gestational diabetes), musculoskeletal disease (including arthritis, rheumatoid arthritis, gout, lupus, or fibromyalgia), chronic respiratory condition (including chronic obstructive pulmonary disease, emphysema, or chronic bronchitis), depression (including depression, major depression, dysthymia, or minor depression), and kidney disease (excluding kidney stones, bladder infection, or incontinence); and count of chronic conditions (0–2, and >2).

Behavioral characteristics that were also considered included smoking status (current, former, never smoker); binge drinking (having four or more drinks on one occasion in the past month); physical activity other than your regular job during the past month (yes/no).^31,34,35^

### Statistical analyses

All analyses, performed using Stata, version 16, were weighted to be representative of the broader population of Latinas in the United States 50–64 years.^22^ The BRFSS weights adjust for the unequal probability of being selected, noncoverage by the survey, and nonresponse. Weights for data from all years were divided by four (*i.e.*, the number of years of survey data available from each jurisdiction). BRFSS sampling strata may vary from year to year, and primary sampling unit (PSU) identifiers are recycled from 1 year's data collection to the next. To avoid treating unrelated observations as coming from related strata or PSUs simply because they were interviewed in different years, we used year-specific stratum and PSU identifiers in all analyses.^36–38^

We conducted separate analyses for breast, cervical, and colorectal cancer screening. First, chi-square tests were used to compare predisposing, enabling, and need factors by whether or not participants were adherent to each cancer screening guideline. Separate weighted Poisson regression models were used to evaluate the association of BMI and cancer screening adherence stratified by place of interview.

Separate weighted Poisson regression models were used to calculate the prevalence ratio (PR) of each cancer screening adherence for Puerto Rico versus rest of United States in each BMI category. In each model, an interaction term between BMI category and place (Puerto Rico, rest of United States) was added to the model to estimate a PR of cancer screening within each BMI category.^39^ We used this approach instead of a regular stratified analysis by each BMI category, because this allows us to have a direct comparison across BMI categories between Hispanic women living in Puerto Rico to those living in the rest of the United States while adjusting for confounders.

For valid statistical inferences, empirical standard errors were used. Unadjusted Poisson models were fitted to estimate the overall PR of cancer screening, and adjusted Poisson models were also fitted to adjust for confounders. In the adjusted models, age, education, number of chronic conditions, health insurance, and year of the survey were included. The addition of other potential confounders did not substantially change the estimates. Crude and adjusted PRs (aPR) and confidence intervals (95% CI) were estimated from these models. From these models, aPR >1 suggested higher prevalence of lack of cancer screening adherence. Conversely, we interpreted aPR <1 to indicate higher prevalence of adherence to each cancer screening.

To understand if there were differences between Latinas living in Puerto Rico in comparison with Latinas living in the rest of United States, a weighted adjusted Wald test evaluated the equality of coefficients.^40,41^ We also performed an analysis evaluating having never versus ever been screened using the same approach described above.

## Results

Our weighted sample represents 3 million Latinas 50 to 64 years of age living in the United States, of whom 11.1% lived in Puerto Rico. Overall, 21.1% were not adherent to breast cancer screening guidelines, 24.2% were not adherent to cervical cancer screening guidelines, and 43.6% were not adherent to colorectal cancer screening guidelines (Table 1).

**Table 1.** Sociodemographic, Clinical, and Health Care Characteristics Among Latinas 50–64 Years of age by Cancer Screening Adherence in the Behavioral Risk Factor Surveillance System, 2012–2018

**Characteristics**	**Breast cancer screening adherence**		**Cervical cancer screening adherence**		**Colorectal cancer screening adherence**	
	**No**	**Yes**	**No**	**Yes**	**No**	**Yes**
Unweighted, *N* (%)	3,625 (22.1)	12,785 (77.9)	3,975 (24.2)	12,435 (75.8)	7,152 (43.6)	9,258 (56.4)
Weighted, *N*	662,235	2,396,589	686,400	2,372,424	1,420,518	1,638,306
**Predisposing**						
Age, years	%					
50–54	42.4	38.4	34.3	40.6	48.4	31.3
55–59	31.8	31.1	32.8	30.8	29.6	32.6
60–64	25.8	30.5	32.9	28.6	22.0	36.0
Marital status						
Married/living together	53.8	58.7	53.3	58.9	55.9	59.2
Divorced/separated	28.5	25.2	26.6	25.7	25.6	26.2
Widowed/never married	17.8	16.1	20.1	15.4	18.5	14.6
Education						
Less than high school	40.7	37.8	41.7	37.4	44.4	33.2
High school	24.4	23.7	25.1	23.5	23.4	24.2
Some college or more	34.9	38.6	33.2	39.1	32.2	42.6
Employment status						
Employed	47.2	46.6	41.0	48.4	48.1	45.6
Homemaker/student	22.1	20.5	21.2	20.8	23.6	18.5
Unemployed/unable to work	23.8	23.8	28.6	22.4	22.5	25.0
Retired	6.9	9.0	9.1	8.4	5.8	10.9
**Enabling**						
Income (<$35,000)	73.1	63.8	72.1	64.1	72.6	60.0
No health care coverage	39.9	17.0	30.6	19.5	33.3	12.2
No personal doctor	35.0	13.0	27.1	15.1	26.6	10.1
Routine checkup (≥12 months or never)	49.6	13.7	37.6	16.8	30.9	13.3
Could not see doctor because of cost	42.2	21.4	34.3	23.5	33.2	19.5
**Need**						
Fair or poor general health status	43.4	41.7	49.6	39.9	42.2	41.9
Self-reported chronic conditions						
Cardiovascular disease	8.6	10.0	10.6	9.4	8.5	10.7
Chronic obstructive pulmonary disease	8.6	7.3	9.6	7.0	6.4	8.6
Arthritis	34.4	40.1	42.0	38.0	31.8	45.0
Asthma	18.1	16.9	20.1	16.3	13.5	20.3
Depressive disorder	23.7	26.0	29.8	24.3	21.9	28.7
Kidney disease	4.5	4.8	5.2	4.6	4.3	5.1
Diabetes	19.8	22.7	24.6	21.3	20.7	23.3
≥2 chronic conditions	29.1	32.4	37.2	30.1	25.6	37.0
**Behavioral characteristics**						
Current smoker	17.7	10.0	15.7	10.5	13.1	10.5
Binge drinker	7.6	6.1	6.8	6.3	5.8	6.9
Nonphysical activity	40.5	35.8	42.7	35.2	38.3	35.6

The Andersen Model of Health Services Use framework was used in this study; the bold indicates the categories included in the framework.

Values presented are weighted according to BRFSS methodology (unweighted *N* = 16,410; weighted *N* = 3,058,824). Missing data: marital status (*n* = 46), employment (*n* = 55): income (*n* = 2,191); no personal doctor (*n* = 52); time last routine (*n* = 125); cost (*n* = 33); health status (*n* = 66); cardiovascular disease (*n* = 6); chronic obstructive pulmonary disease (*n* = 63); arthritis (*n* = 96); asthma (*n* = 30); depression (*n* = 51); kidney (*n* = 58); diabetes (*n* = 31); smoke (*n* = 57); binge (*n* = 214); physical activity (*n* = 26).

BRFSS, Behavioral Risk Factor Surveillance System.

Latinas who were not adherent to breast cancer screening guidelines were younger (42.4% not adherent vs. 38.4% adherent were 50–54 years old) and less educated (40.7% not adherent vs. 37.8% adherent had completed less than high school) than Latinas who were ever screened for breast cancer.

In terms of enabling factors, a higher proportion of Latinas never screened for breast cancer reported income less than $35,000 (73.1% not adherent vs. 63.8% adherent), lacked health care coverage (39.9% not adherent vs. 17.0% adherent), had no personal doctor (35.0% not adherent vs. 13.0% adherent), did not have a routine checkup (49.6% not adherent vs. 13.7% adherent), and could not see a doctor because of cost (42.2% not adherent vs. 21.4% adherent) relative to Latinas who were adherent to breast cancer screening.

Latinas adherent to breast cancer screening were more likely to have a self-reported chronic condition than Latinas who reported no adherence to breast cancer screening (29.1% not adherent vs. 32.4% adherent had ≥2 chronic conditions). Similar findings were observed for cervical and colorectal screening tests. However, Latinas who did not adhere to cervical cancer screening were more likely to have self-reported chronic conditions than Latinas who were adherent to cervical cancer screening (37.2% not adherent vs. 30.1% adherent had ≥2 chronic conditions, Table 1).

**FIG. 1.**
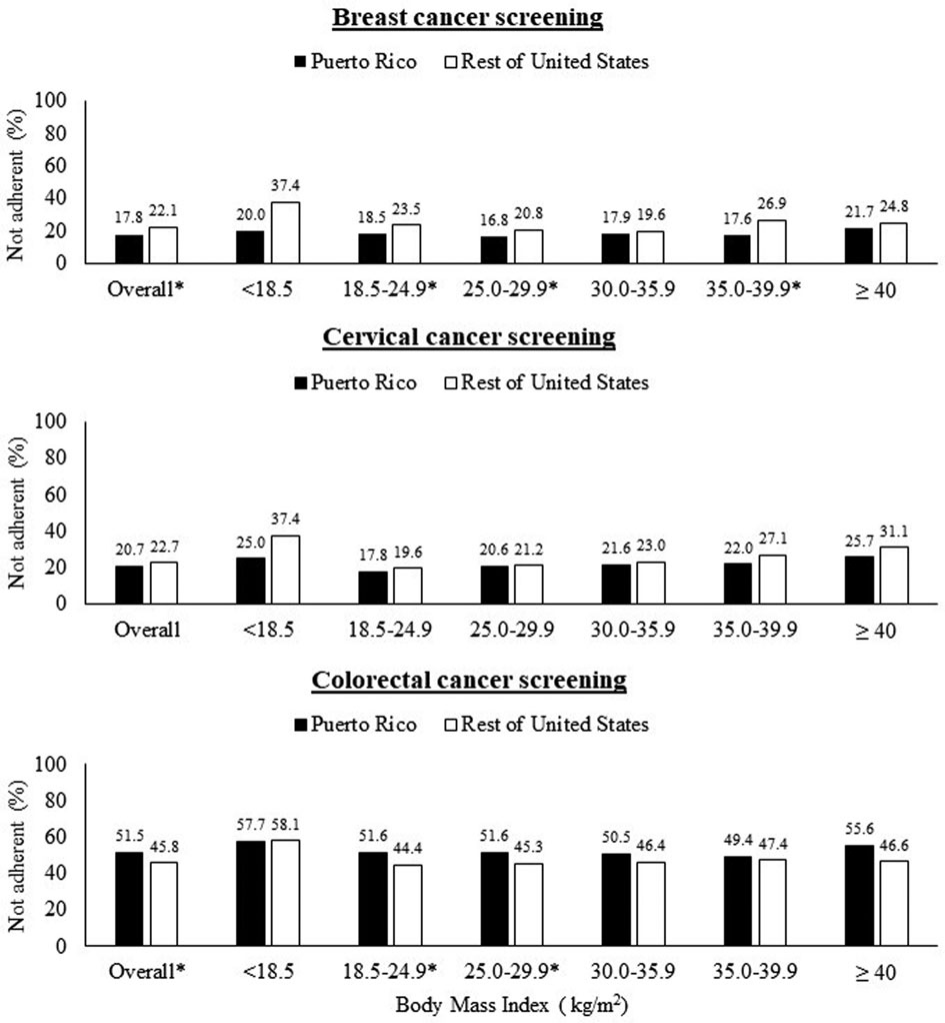
Percent of Latinas 50–64 years of age adherent to screened by cancer type (breast, cervical, colorectal cancer), body mass index, and place (Puerto Rico and rest of the United States): BRFSS 2012–2018. BRFSS, Behavioral Risk Factor Surveillance System.

Overall, a lower percent of Latinas living in Puerto Rico reported not having been adherent to breast cancer screening (17.8% vs. 22.1%) compared with Latinas living in the United States, whereas a higher percent of Latinas living in Puerto Rico reported not having been adherent to colorectal cancer screening (51.5% vs. 45.8%) compared with Latinas living in the United States. No overall difference was observed for cervical cancer screening between Latinas living in Puerto Rico and the rest of the United States (Fig. 1).

However, after adjusting for other confounders, Latinas living in Puerto Rico were 37% (95% CI: 1.30–1.46) more likely to have not been adherent for colorectal cancer screening compared with Latinas living in the United States. No association was found for breast and cervical cancer screening (Table 2).

**Table 2.** Association Between Place of Interview, and Body Mass Index and Lack of Adherence to Cancer Screening Guidelines Among Latinas 50–65 Years of Age (2012–2018)

**Cancer screening**	**Puerto Rico vs. rest of United States**	
	**Crude PR (95% CI)**	**aPR (95% CI)^a^**
Breast cancer—not adherent vs. adherent		
Overall	0.80 (0.72–0.90)	1.02 (0.91–1.15)
BMI, kg/m^2^		
<18.5	0.53 (0.26–1.11)	0.70 (0.35–1.40)
18.5–24.9	0.79 (0.63–0.98)	0.98 (0.78–1.22)
25.0–29.9	0.81 (0.67–0.97)	1.04 (0.86–1.25)
30.0–34.9	0.91 (0.72–1.16)	1.16 (0.92–1.47)
35.0–39.9	0.66 (0.45–0.96)	0.84 (0.57–1.22)
≥40.0	0.87 (0.56–1.35)	1.08 (0.69–1.68)
Cervical cancer—not adherent vs. adherent		
Overall	0.91 (0.82–1.01)	1.01 (0.91–1.12)
BMI, kg/m^2^		
<18.5	0.67 (0.34–1.32)	0.75 (0.38–1.49)
18.5–24.9	0.91 (0.72–1.14)	0.98 (0.78–1.24)
25.0–29.9	0.97 (0.82–1.15)	1.08 (0.91–1.27)
30.0–34.9	0.94 (0.76–1.16)	1.01 (0.82–1.25)
35.0–39.9	0.81 (0.59–1.11)	0.89 (0.65–1.23)
≥40.0	0.83 (0.57–1.20)	0.95 (0.65–1.40)
Colorectal cancer—not adherent vs. adherent		
Overall	1.12 (1.06–1.19)	1.37 (1.30–1.46)
BMI, kg/m^2^		
<18.5	0.99 (0.67–1.48)	1.33 (0.87–2.03)
18.5–24.9	1.16 (1.04–1.30)	1.39 (1.24–1.55)
25.0–29.9	1.14 (1.04–1.25)	1.40 (1.29–1.53)
30.0–34.9	1.09 (0.97–1.22)	1.36 (1.22–1.52)
35.0–39.9	1.04 (0.86–1.26)	1.27 (1.05–1.54)
≥40.0	1.19 (0.97–1.47)	1.38 (1.12–1.70)

Values presented are weighted according to BRFSS methodology. The reference group are Latinas in the rest of United States in each. The estimates were obtained from a model, including interaction term between BMI category and place (Puerto Rico and rest of United States).

^a^ Adjusted for age, education, number of chronic conditions, health insurance, and year of the survey. Overall estimated are also adjusted for BMI.

BMI, body mass index; CI, confidence interval; aPR, adjusted prevalence ratio.

We evaluated the association between BMI and lack of adherence to cancer screening stratified by place of interview (Supplementary Table S1). We found that, regardless of place, Latinas with BMI ≥40.0 kg/m^2^ were more likely to lack adherence to cervical cancer screening than women with BMI 18.5–24.9 kg/m^2^. Latinas living in the rest of United States who report a BMI <18.5 kg/m^2^, were 72% more likely to lack adherence to cervical cancer screening than women who report BMI 18.5–24.9 kg/m^2^. No association was found between BMI, and breast and colorectal cancer screening (Supplementary Table S1).

In crude prevalence estimates across BMI categories, a lower percent of Latinas living in Puerto Rico were not adherent to breast and cervical cancer screening compared with Latinas living in the United States of the same BMI range (Fig. 1 and Table 2). A lower percent of Latinas living in Puerto Rico who reported a BMI lower than 18.5 kg/m^2^ were not adherent to breast (20.0% vs. 37.4%) and cervical (25.0% vs. 37.4%) screening compared with Latinas living in the United States of the same BMI range (Fig. 1), but their crude PRs were not statistically significant (PR: 0.53 [95% CI: 0.26–1.11] for breast cancer adherence; PR: 0.67 [95% CI: 0.34–1.32] for cervical cancer adherence; Table 2).

In the crude PR estimates, a significantly lower percent of Latinas living in Puerto Rico who report a BMI of 18.5–24.9 and 35.0–39.9 kg/m^2^ have not been adherent to breast cancer screenings compared with Latinas living in the United States of the same BMI range; 0.791 (95% CI: 0.63–0.98) in BMI 18.5–24.9 kg/m^2^ and 0.66 (95% CI: 0.45–0.96) in BMI 35.0–39.9 kg/m^2^.

For colorectal cancer screening, the proportion of Latinas not adherent living in Puerto Rico ranged from 49.4% to 57.7% across BMI categories, whereas the proportion of Latinas never screened living in other parts of the United States ranged from 44.4% to 58.1% across BMI categories (Fig. 1), with aPR ranges from 1.27 to 1.40 (Table 2).

After accounting for other confounders, the overall association between BMI with breast and cervical cancer screening adherence was not different between Latinas living in Puerto Rico and Latinas living in the United States. We found differences in the association between BMI and lack of adherence to colorectal cancer screening between Latinas living in the United States and Puerto Rico. Specifically, Latinas living in Puerto Rico who had BMI greater than 18.5 kg/m^2^ were more likely to have not been adherent with colorectal cancer screening guidelines than Latinas with the same BMI living in the rest of the United States.

For those whose BMI was greater than 40 kg/m^2^, Latinas living in Puerto Rico were 38% (95% CI: 1.12–1.70) more likely to lack adherence to colorectal cancer screening guidelines than Latinas living in the rest of the United States (Table 2). In an additional analysis evaluating having never versus ever been screened, we found that for those whose BMI was greater than 40 kg/m^2^, Latinas living in Puerto Rico were 70% (95% CI: 1.35–2.14) more likely to never have been screened for colorectal cancer than Latinas in the rest of the United States (Supplementary Table S2).

## Discussion

Overall, we found 77.9% and 75.8% of Latinas in Puerto Rico and the rest of the United States were adherent with breast and cervical cancer screening guidelines, respectively. Whereas 43.6% of Latinas were not adherent with colorectal cancer screening guidelines. The association between body size and cancer screening utilization among Latinas differed by cancer screening type and by location (Puerto Rico vs. those living in the rest of the United States). For colorectal cancer and breast cancer, an association between higher BMI and nonadherence cancer screening in Latinas was not supported by our data.

However, regardless of place, Latinas with higher BMIs were more likely to lack adherence to cervical cancer screening guidelines. For colorectal cancer screening, Latinas with larger body size as indicated by higher BMI living in Puerto Rico were more likely to not be adherent with colorectal cancer screening guidelines than Latinas living in the rest of the United States.

In Puerto Rico and the rest of the United States, Latinas with higher BMI appeared to have higher prevalence of breast cancer screening adherence than Latinas living in the rest of the United States; however, estimates in most higher BMI categories lacked precision. Previous studies have shown a decrease in breast cancer screening rates in women whose BMI is >25 kg/m^2^.^14,42^ However, similar to our findings, an analysis using the 2016 BRFSS found no association between obesity and adherence to breast cancer screening.^43^

It is possible that weight-related facility-level barriers around breast cancer screening have been reduced, possibly due to an assumption that people in larger bodies have health problems and are more likely to get cancer screening than people with smaller body sizes.^44,45^

For cervical cancer, an association between larger body size and cancer screening adherence in Latinas was observed, regardless of the place of the interview. Similar to our findings, a study among 536 Latinas living in Puerto Rico, 21 to 64 years of age found than Latinas living in Puerto Rico with BMI 40 kg/m^2^ or higher were less likely to not be adherent to cervical cancer screening guidelines.^46^ In contrast, a previous study using data from the 2000 National Health Interview Survey found no association between BMI and cervical cancer screening among Hispanics.^47^ In our study, we included women 50–64 years of age. It is possible that this association could be different among younger women. Future studies should evaluate this association among different age groups.

It is unclear why women with lower BMI may be at risk for not receiving cervical cancer screening tests, but it has been suggested that these results could be related to an illness that precluded the use of these screenings, a general reluctance to seek care, and fear of being questioned about having an eating disorder.^42^ Also it is possible that people with smaller body sizes are often not considered as high risk as people with larger bodies regardless of their actual health metrics. Doctors may be more likely to think that any health problems are less severe because their patients look healthy.^48^

Four in 10 Latinas had never been screened for colorectal cancer. Previous studies have documented that Hispanics have lower colorectal screening rates across most U.S. states/territories.^49,50^ It has been suggested that this disparity could be related to multiple factors, including low educational attainment, low income, and lack of access to health care services.^49^

Additionally, previous studies have shown that Hispanics who were employed or who reported excellent or good health may be less likely to pursue health care in general or prioritize preventive services, potentially due to employers not offering paid time away from work to participate in health visits and preventive services.^51^ We found differences in the association between BMI categories and colorectal cancer screening between Latinas living in the United States and Puerto Rico.

For those with BMI greater than 18.5 kg/m^2^, Latinas living in Puerto Rico were more likely to lack adherence to colorectal cancer screening guidelines than Latinas living in the rest of the United States. Previous studies investigating BMI and colorectal cancer screening have been inconsistent. Some studies found that adults with obesity were less likely to have received a colorectal cancer screening test,^17,52^ while others reported that overweight and obese adults were both slightly more likely to have received colorectal cancer screening compared with their healthy weight counterparts,^53^ and other studies found no association between colorectal cancer screening and BMI.^54^ None of these studies presented results specifically among Hispanics.

Our study documented less frequent cancer screening practices among underweight Latinas. Previous analyses using the 2016 BRFSS showed that underweight women were less likely to be adherent to breast and cervical cancer screening than women with BMI between 18.5 and 24.9 kg/m^2^, but no association was observed among colorectal cancer screening.^55^ While results from Latinas in Puerto Rico are imprecise, the percent of underweight Latinas living in the rest of the United States who lacked adherence to cancer screening guidelines appeared higher than those living in Puerto Rico.

Limited studies have evaluated the association between underweight and concordance with cancer screening guidelines, because most studies excluded or combined BMI categories in the lower range. These limitations in the literature may have masked a disparity among women with lower BMI who may be facing different and distinct health care issues. Additional research is needed to understand barriers to screening for those with very low BMI.

Puerto Ricans' life experiences and access to care may be different from other Hispanic/Latino subgroups.^56^ For 2012, nearly 45% of the Puerto Rican population were covered by the Puerto Rico Government Health Plan, of which 5% were dual eligible (Medicaid and Medicare).^57^ The Puerto Rico Government Health Plan covers all preventive services, including breast, cervical, and colorectal cancer screenings.

Hispanics from the rest of United States come from different nationalities and have different experiences with health care, for example Hispanic/Latinos from underdeveloped countries have limited access to health care in the United States and immigrants may experience the same barriers as in their countries of origin.^58^ Latinas may lack money for treatment, or may experience challenges in taking time off from work for such tests. Undocumented Latinos may face challenges as they may fear losing their job and/or getting deported if they request time off for health care appointments.^58^

A public assistance program for undocumented patients and other noncitizens in the United States exists; however, the system is difficult to navigate and may discourage Latinas to get preventive services.^59^ In addition, many Latino/Hispanic countries do not have robust cancer screening programs, and some tests are less commonly performed in Hispanic/Latino countries than in the United States.^60^ In addition, patients may be more likely to seek medical attention only when symptoms arise.^58^ Language barriers are a concern; non-English-speaking Latinas are less likely to access general preventive services.^61^ Other barriers to receiving preventive care include stigma or fear about the importance of the screening tests, and lack of transportation.^62^

These results should be interpreted considering several limitations. First, given that the BRFSS is a cross-sectional study, the temporal sequence between cancer screening and BMI is unclear. Data for the BRFSS were collected by phone interview, and data on cancer screening utilization, weight, and height were self-reported.

The BRFSS does not ask respondents to specify the reason why these tests were conducted (*e.g.*, for screening vs. diagnostic reasons). As a result, we could only discern if the respondent had a particular test and when it was last administered, and by default, we likely included tests done for reasons other than asymptomatic screening. For this reason, it is possible that our analysis overestimates screening rates. BRFSS data do not provide consistent information regarding Latina subgroups (*e.g.*, Mexican, Cuban, Puerto Ricans) or immigration status.

Latinas are a heterogeneous population, and a more granular analysis between subgroups may reveal markedly different results. However, we were able to evaluate differences in the association between BMI categories and cancer screening comparing Hispanics living in Puerto Rico and the United States. Latinas living in Puerto Rico could also be from other nationalities like the Dominican Republic, although we were unable to discern those differences in this analysis. Latinas living in the United States tend to have more diverse nationalities, including higher proportions from Mexico and Puerto Rico.

## Conclusion

In this study of Latinas living in Puerto Rico and the rest of the United States, we found that Latinas' self-reported BMI was associated with cancer screening utilization in certain subgroups. Notable differences were observed among Latinas living in Puerto Rico, who were less likely to be adherent to colorectal cancer screening than Latinas living in the United States regardless of BMI. Given the importance of receiving regular preventive cancer screening tests in accordance with USPSTF guidelines, it is crucial that public health and clinical professionals work to improve screening rates among Latinas.

The role of body size in understanding utilization of cancer screening among Latinas differs in women living in Puerto Rico versus those living in the rest of the United States and varies across cancer type. Understanding the unique barriers to receiving screening for each cancer type is important for the development of adequate culturally adapted interventions among Latina women.

## Supplementary Material

Supplemental data

Supplemental data

Supplemental data

## Abbreviations Used

aPR: adjusted prevalence ratios
BMI: body mass index
BRFSS: Behavioral Risk Factor Surveillance System
CI: confidence intervals
COPD: chronic obstructive pulmonary disease
FIT: fecal immunochemical test
FOBT: fecal occult blood test
hrHPV: high-risk human papilloma virus test
PSU: primary sampling unit
USPSTF: US Preventive Services Task Force
