# Supplementary material for: Association Between Body Mass Index and Cancer Screening Adherence Among Latinas in the United States and Puerto Rico
Source: Womens Health Rep (New Rochelle). 2022 May 31;3(1):552–62. doi: 10.1089/whr.2021.0153 (PMC10122236; doi:10.1089/whr.2021.0153)
Supplement: Supplemental data [file Suppl_FigS1.docx]

**Supplemental figure 1.** Inclusion and exclusion criteria
