# Supplementary material for: Association Between Body Mass Index and Cancer Screening Adherence Among Latinas in the United States and Puerto Rico
Source: Womens Health Rep (New Rochelle). 2022 May 31;3(1):552–62. doi: 10.1089/whr.2021.0153 (PMC10122236; doi:10.1089/whr.2021.0153)
Supplement: Supplemental data [file Suppl_TableS1.docx]

**Supplemental Table 1.** Association between body mass index and lack of adherence to cancer screening stratified by place of interview among Latinas aged 50-65: BRFSS 2012-2018

| **Cancer screening** | **Puerto Rico** | | **Rest of United States** | |
| --- | --- | --- | --- | --- |
|  | Crude Prevalence Ratio (PR) (95% Confidence Interval (CI)) | Adjusted PR  (95% CI)* | Crude PR (95% CI) | Adjusted PR  (95% CI)* |
| **Breast cancer- Not adherent vs adherent** | | |  |  |
| **Body mass index** |  |  |  |  |
| *<18.5 kg/m^2^* | 1.08 (0.58-2.03) | 0.99 (0.53-1.84) | 1.59 (1.02-2.48) | 1.44 (0.95-2.19) |
| *18.5-24.9 kg/m^2^* | Reference | Reference | Reference | Reference |
| *25.0-29.9 kg/m^2^* | 0.91 (0.72-1.15) | 0.90 (0.71-1.13) | 0.88 (0.74-1.05) | 0.87 (0.74-1.03) |
| *30.0-34.9 kg/m^2^* | 0.97 (0.74-1.27) | 1.00 (0.77-1.30) | 0.83 (0.69-1.01) | 0.84 (0.70-1.01) |
| *35.0-39.9 kg/m^2^* | 0.95 (0.66-1.37) | 1.01 (0.71-1.45) | 1.14 (0.89-1.48) | 1.18 (0.91-1.53) |
| *≥40.0 kg/m^2^* | 1.17 (0.76-1.80) | 1.29 (0.85-1.96) | 1.06 (0.83-1.35) | 1.09 (0.85-1.40) |
| **Cervical cancer- Not adherent vs adherent** | | |  |  |
| **Body mass index** |  |  |  |  |
| *<18.5 kg/m^2^* | 1.40 (0.79-2.47) | 1.28 (0.71-2.29) | 1.91 (1.22-2.99) | 1.72 (1.09-2.71) |
| *18.5-24.9 kg/m^2^* | Reference | Reference | Reference | Reference |
| *25.0-29.9 kg/m^2^* | 1.16 (0.92-1.45) | 1.14 (0.91-1.43) | 1.08 (0.91-1.28) | 0.89 (0.89-1.24) |
| *30.0-34.9 kg/m^2^* | 1.21 (0.94-1.56) | 1.19 (0.93-1.53) | 1.18 (0.98-1.41) | 1.11 (0.93-1.34) |
| *35.0-39.9 kg/m^2^* | 1.23 (0.90-1.69) | 1.24 (0.90-1.70) | 1.38 (1.10-1.75) | 1.29 (1.02-1.62) |
| *≥40.0 kg/m^2^* | 1.44 (0.98-2.10) | 1.51 (1.04-2.21) | 1.59 (1.27-1.98) | 1.42 (1.12-1.79) |
| **Colorectal cancer- Not adherent vs adherent** | | |  |  |
| **Body mass index** |  |  |  |  |
| *<18.5 kg/m^2^* | 1.18 (0.82-1.52) | 1.09 (0.78-1.52) | 1.31 (0.99-1.72) | 1.14 (0.87-1.49) |
| *18.5-24.9 kg/m^2^* | Reference | Reference | Reference | Reference |
| *25.0-29.9 kg/m^2^* | 1.00 (0.90-1.11) | 0.99 (0.90-1.09) | 1.02 (0.92-1.72) | 0.99 (0.90-1.09) |
| *30.0-34.9 kg/m^2^* | 0.98 (0.87-1.10) | 0.98 (0.89-1.10) | 1.02 (0.92-1.13) | 1.03 (0.93-1.15) |
| *35.0-39.9 kg/m^2^* | 0.96 (0.81-1.12) | 0.98 (0.84-1.15) | 1.04 (0.91-1.24) | 1.10 (0.95-1.28) |
| *≥40.0 kg/m^2^* | 1.08 (0.98-1.29) | 1.10 (0.92-1.31) | 1.05 (0.90-1.23) | 1.12 (0.96-1.31) |
