# Supplementary material for: Association Between Body Mass Index and Cancer Screening Adherence Among Latinas in the United States and Puerto Rico
Source: Womens Health Rep (New Rochelle). 2022 May 31;3(1):552–62. doi: 10.1089/whr.2021.0153 (PMC10122236; doi:10.1089/whr.2021.0153)
Supplement: Supplemental data [file Suppl_TableS2.docx]

**Supplemental Table 2.** Association between body mass index and lack of cancer screening among Latinas by cancer type (2012-2018).

| **Cancer screening** | **Puerto Rico vs Rest of United States** | |
| --- | --- | --- |
|  | Crude Prevalence Ratio (PR) (95% Confidence Interval (CI)) | Adjusted PR  (95% CI)* |
| **Breast cancer- Never screened vs ever screened** | | |
| **Body mass index** |  |  |
| *<18.5 kg/m^2^* | 0.22 (0.05-0.91) | 0.34 (0.09-1.37) |
| *18.5-24.9 kg/m^2^* | 0.94 (0.54 -1.62) | 1.28 (0.73-2.23) |
| *25.0-29.9 kg/m^2^* | 0.64 (0.41-0.99) | 0.93 (0.59-1.45) |
| *30.0-34.9 kg/m^2^* | 0.76 (0.42-1.38) | 1.09 (0.60-2.01) |
| *35.0-39.9 kg/m^2^* | 0.78 (0.33-1.82) | 1.13 (0.48-2.61) |
| *≥40.0 kg/m^2^* | 0.59 (0.12-2.76) | 0.87 (0.18-4.10) |
| **Cervical cancer- Never screened vs ever screened** | | |
| **Body mass index** |  |  |
| *<18.5 kg/m^2^* | 0.47 (0.12-1.83) | 0.56 (0.12-2.28) |
| *18.5-24.9 kg/m^2^* | 1.60 (1.01-2.53) | 1.79 (1.13-2.82) |
| *25.0-29.9 kg/m^2^* | 0.54 (0.35-0.83) | 0.63 (0.40-0.98) |
| *30.0-34.9 kg/m^2^* | 0.86 (0.48-1.53) | 0.97 (0.55-1.73) |
| *35.0-39.9 kg/m^2^* | 0.70 (0.31-1.59) | 0.79 (0.35-1.81) |
| *≥40.0 kg/m^2^* | 0.51 (0.20-1.30) | 0.62 (0.24-1.61) |
| **Colorectal cancer- Never screened vs ever screened** | | |
| **Body mass index** |  |  |
| *<18.5 kg/m^2^* | 0.77 (0.49-1.21) | 1.11 (0.69-1.80) |
| *18.5-24.9 kg/m^2^* | 1.21 (1.06-1.38) | 1.51 (1.32-1.71) |
| *25.0-29.9 kg/m^2^* | 1.12 (1.01-1.24) | 1.44 (1.30-1.59) |
| *30.0-34.9 kg/m^2^* | 1.15 (1.02-1.31) | 1.50 (1.33-1.70) |
| *35.0-39.9 kg/m^2^* | 1.12 (0.90-1.39) | 1.41 (1.14-1.73) |
| *≥40.0 kg/m^2^* | 1.41 (1.10-1.79) | 1.70 (1.35-2.14) |

Values presented are weighted according to BRFSS methodology. The estimates were obtained from a model including interaction term between BMI category and place (Puerto Rico and rest of United States).

*Adjusted for age, education, number of chronic conditions, health insurance and year of the survey.

Values presented are weighted according to BRFSS methodology (Unweighted N = 17,136; weighted N = 3,217,522), the sample size was different in this analysis because we did not exclude participant who failed to respond the question related to the last time the test was performed.
